# Circadian Rhythms in Environmental Health Sciences

**DOI:** 10.1007/s40572-020-00285-2

**Published:** 2020-07-13

**Authors:** Jacqueline M. Leung, Micaela E. Martinez

**Affiliations:** Department of Environmental Health Sciences, Columbia University, 630 West 168th Street, Room 16-421C, New York, NY USA

**Keywords:** Circadian rhythms, Environmental health, Biomarkers, DNA methylation, Asthma, Breast cancer

## Abstract

**Purpose of Review:**

This review aims to explore how circadian rhythms influence disease susceptibility and potentially modify the effect of environmental exposures. We aimed to identify biomarkers commonly used in environmental health research that have also been the subject of chronobiology studies, in order to review circadian rhythms of relevance to environmental health and determine if time-of-day is an important factor to consider in environmental health studies. Moreover, we discuss opportunities for studying how environmental exposures may interact with circadian rhythms to structure disease pathology and etiology.

**Recent Findings:**

In recent years, the study of circadian rhythms in mammals has flourished. Animal models revealed that all body tissues have circadian rhythms. In humans, circadian rhythms were also shown to exist at multiple levels of organization: molecular, cellular, and physiological processes, including responding to oxidative stress, cell trafficking, and sex hormone production, respectively. Together, these rhythms are an essential component of human physiology and can shape an individual’s susceptibility and response to disease.

**Summary:**

Circadian rhythms are relatively unexplored in environmental health research. However, circadian clocks control many physiological and behavioral processes that impact exposure pathways and disease systems. We believe this review will motivate new studies of (i) the impact of exposures on circadian rhythms, (ii) how circadian rhythms modify the effect of environmental exposures, and (iii) how time-of-day impacts our ability to observe the body’s response to exposure.

## Introduction

Circadian rhythms exist in species throughout the tree of life, from single-cell organisms (e.g., cyanobacteria and Trypanosomes) to humans. Circadian rhythms drive 24-h cycles in physiology and behavior and evolved in response to predictable changes in Earth’s environment that occur around the day-night cycle [1]. By structuring biological processes into 24-h cycles, the circadian system enables organisms to synchronize their internal biology with their external environment and optimally time activities to maximize fitness and survival [2]. For example, circadian rhythms in human metabolic processes are believed to be evolutionarily advantageous for structuring metabolic activity according to the human diel lifestyle of nighttime sleep, daytime wake activity, and daytime food intake [3].

In mammals, including humans, circadian rhythms are present in nearly all tissues and cells in the body [4•]. The master circadian clock, in the suprachiasmatic nucleus (SCN) of the hypothalamus, is composed of clock genes. Clock genes include several positive genes (i.e., CLOCK, BMAL1) and negative genes (i.e., PER, CRY) that are organized through cell-autonomous transcription-translation feedback loops [5]. The CLOCK:BMAL1 heterodimer binds to DNA and drives the rhythmic transcription of PER and CRY, whose protein products in turn inhibit the activity of the CLOCK:BMAL1 complex. Together, these factors regulate the downstream rhythmic expression of thousands of clock-controlled genes that generate oscillations in physiology and behavior. Light entrains the SCN and keeps clock genes appropriately transcriptionally aligned with the day-night cycle [6]. The SCN then helps to entrain peripheral clocks throughout the body via neuronal signals, hormones, metabolites, and changes in body temperature [7].

The maintenance of circadian rhythms is critical to human health and survival. There are deleterious perturbations to circadian rhythms that include circadian disruption, physical and social jetlag (social jetlag occurs due to different sleep/wake schedules kept on work versus non-work days), and circadian misalignment. Circadian disruption occurs due to a lack of synchronization between body clocks and the day-night cycle and can cause misalignment among the various circadian clocks present throughout the body [8]. Well-known causes of circadian disruption/misalignment include various occupational and household environmental factors such as shift work [9], jetlag [10], and exposure to artificial light-at-night [11]. Overall, circadian disruption, whether due to jetlag or shift work, is mainly driven by light exposure during the body’s biological night (i.e., subjective night) [12•]. There is a growing literature demonstrating that chronic circadian disruption can contribute to the development of diseases, including asthma, cancer, metabolic syndromes, and cardiovascular disease [3, 13, 14]. Disease risk associated with circadian disruption led the American Medical Association in 2012 to adopt a policy statement concluding that “The natural 24-hour cycle of light and dark helps maintain precise alignment of circadian biological rhythms,…Pervasive use of nighttime lighting disrupts these endogenous processes and creates potentially harmful health effects and/or hazardous situations with varying degrees of harm” [12•, 15], and, in 2007, the World Health Organization’s International Agency for Research on Cancer (IARC) classified shift work that involves circadian disruption as a probable human carcinogen [16].

**Fig. 1:**
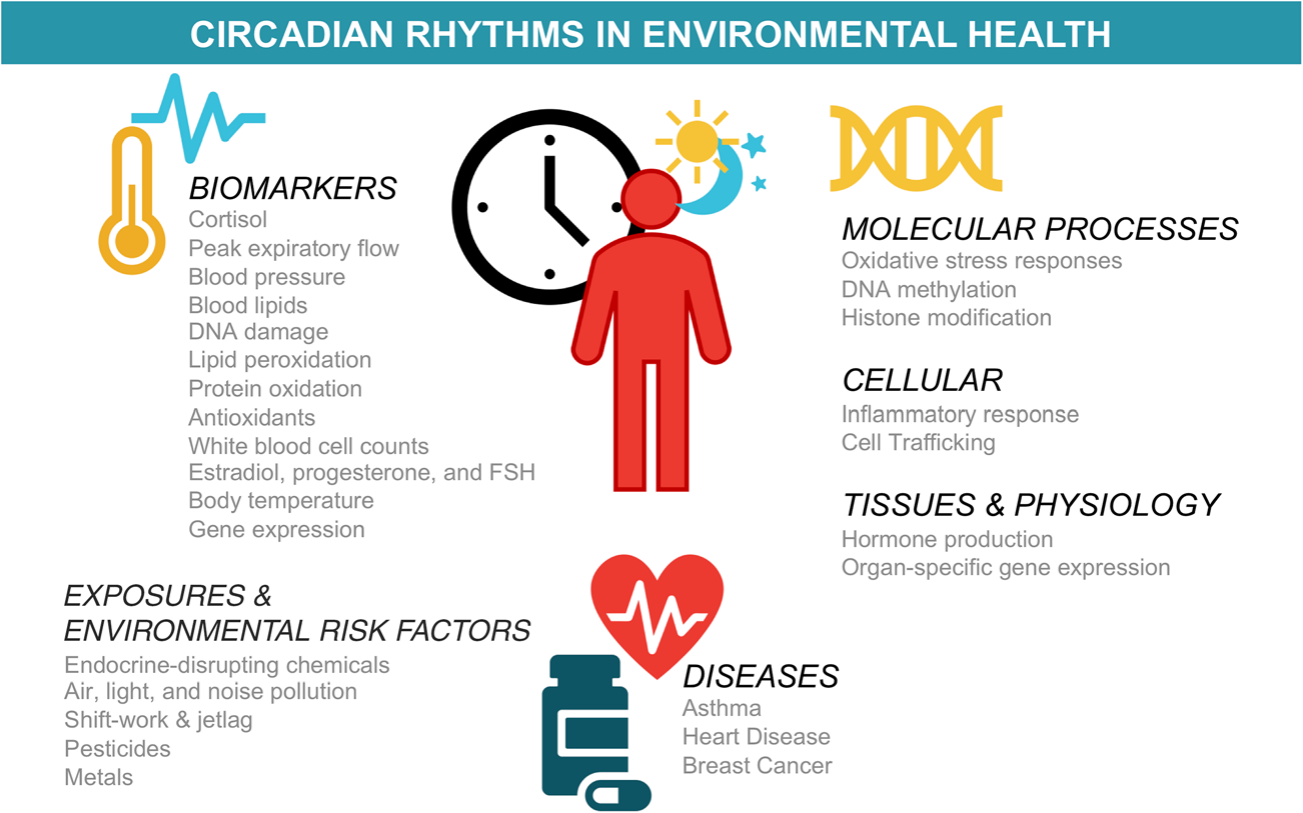
Circadian rhythms in environmental health. This figure illustrates biomarkers, and molecular, cellular, tissue, and physiological processes that have been studied in chronobiology and are also targets of environmental exposures and/or biomarkers deployed in studies of environmental determinants of health

There is a growing literature demonstrating that many diseases, including asthma and heart attack, have important circadian features, sometimes with the incidence and/or severity varying across the 24-h period [1, 8, 13, 17]. For example, the frequency of sudden cardiac death peaks in the morning [18], with a 40% increased risk of heart attack between 6 a.m. and noon [17], and the severity of asthma symptoms often worsens at night [19]. A better understanding of how the circadian system influences disease—particularly how circadian rhythms modify the body’s response to the environment and exposures—could provide new insights into disease etiology and lead to new strategies for prevention and treatment. Here, we aim to identify chronobiology studies that may inform environmental health research relating to exposures such as endocrine-disrupting chemicals, air pollution, noise/light pollution, and environmental determinants of chronic disease. We discuss circadian impacts on the pathophysiology and/or treatment of asthma, heart disease, and breast cancer because these are diseases for which both chronobiology and environmental health research exist to date, and we explore how circadian rhythms influence biological targets of environmental exposures and biomarkers that are commonly used in the study of environmental exposures (Fig. 1). As a future perspective, we also discuss ways in which studies of environmental exposures and environmental determinants of health can deploy circadian and time-dependent sampling to study how circadian rhythms may act as a modifier of disease.

## Circadian Rhythms and Disease

In reviewing the chronobiology literature, we identified three diseases—asthma, heart disease, and breast cancer—that have been the subject of chronobiology studies and have also been studied in environmental health in relation to environmental exposures and/or environmental determinants of health. The intersection of environmental health and chronobiology likely go beyond these diseases, but this is the state of the field to date.

The incidence and severity of many diseases varies across the day and night [1, 8]. We describe how circadian rhythms in underlying biological processes can shape the overall presentation of disease (e.g., as in asthma and heart disease) and how circadian disruption due to occupational environmental exposures may elevate risk of disease (e.g., as in breast cancer).

### Asthma

One of the major diseases known to have circadian variation is asthma. Asthma is a chronic, inflammatory disease of the respiratory system associated with exposure to environmental factors such as allergens, smoke, and air pollution [20]. Importantly, asthma symptoms show pronounced time-of-day variation in occurrence and severity. For instance, exacerbated asthma symptoms occur primarily at night and peak around 04:00 a.m. [19]. The majority of asthma deaths also occur between midnight and 06:00 a.m. [21]. The elevated nighttime incidence of asthma exacerbations has been associated with circadian changes in pulmonary function, including a nocturnal increase in airway inflammation, bronchial responsiveness, and eosinophils (i.e., cells of the immune system associated with asthma and allergy) in bronchial tissue [22]. The nocturnal increase in eosinophils is correlated with an increase in lung airway obstruction. Animal models of asthma have indicated that the expression of the clock gene BMAL1 plays a role in eosinophil trafficking and the production of the cytokine IL-5 [23], which is a target of multiple asthma drugs and has been shown to be under epigenetic regulation [24].

**Fig. 2:**
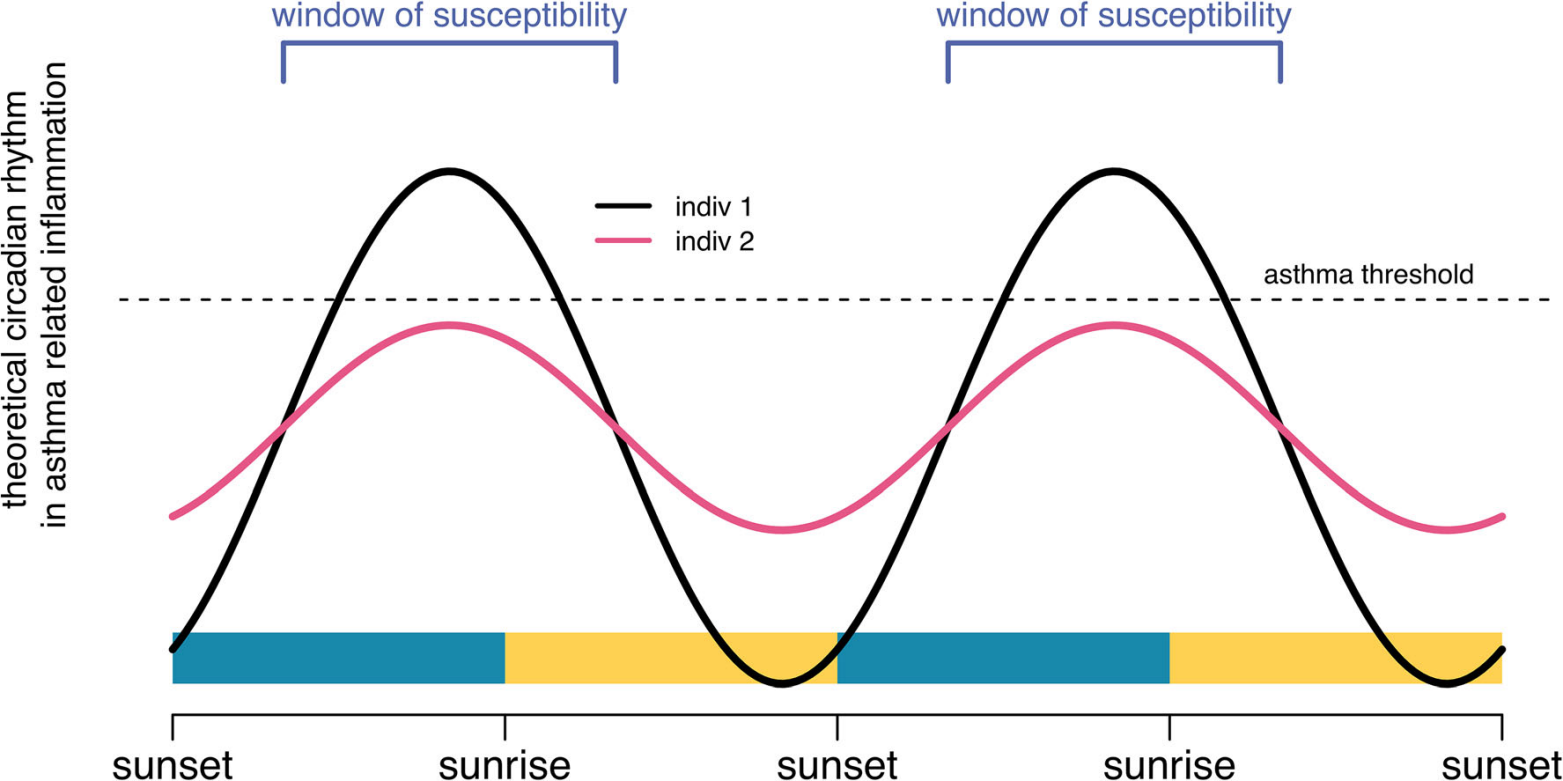
Hypothetical mechanism by which circadian rhythms in inflammation affect asthmatic symptoms. The figure shows a theoretical threshold of inflammation in bronchial tissue, above which an individual may clinically present with asthma. First, we hypothesize high amplitude circadian rhythms (e.g., individual 1) may predispose individuals to asthma. Second, we hypothesize that time-of-day effects on asthma are due to circadian rhythms opening up a window of susceptibility that modifies the effect of environmental exposures, resulting in a higher likelihood of asthma exacerbations

Not only do experimental and clinical studies suggest the pathology of asthma is impacted by circadian rhythms, these rhythms are likely important for disease manifestation and treatment. For instance, individuals with asthma often have circadian rhythms with an exaggerated amplitude. Fluctuations in airway obstruction, as measured by peak expiratory flow (PEF) over 24 h, were found to be synchronized between healthy and asthmatic individuals, with both having more obstruction occurring at night. However, the amplitude of PEF variation was 50.9% greater in asthma patients compared to controls [25]. From a pathology perspective, we hypothesize that the amplitude of an individual’s circadian rhythm in PEF could in part predispose them to asthma attacks and/or modify severity when an attack is triggered, for instance by environmental exposures. Figure 2 illustrates how circadian amplitude and time-of-day (i.e., circadian phase) could physiologically position individuals such that they are susceptible to an asthma exacerbation. Since circadian rhythms are present in utero, with humans having circadian rhythms as early as 30 weeks of gestation [26], they have the potential to play a role in diseases such as asthma that may stem from developmental periods.

### Heart Disease

Exposure to environmental pollutants, such as pesticides, tobacco smoke, and fine particulate matter, play an important role in the development and severity of cardiovascular disease [27, 28]. These exposures impact aspects of physiology that are known to affect cardiovascular risk, including changes in blood pressure, endothelial function, blood lipids, and blood coagulation markers [29]. Importantly, these physiological aspects also display time-dependent oscillations throughout the day-night cycle [30]. For instance, blood pressure surges upon waking [31], and vascular endothelial function is impaired in the morning [32]. Circadian variation in these factors parallel the pattern of adverse cardiovascular events, including myocardial infarction [33, 34], stroke [35], ventricular arrhythmia [36], and sudden cardiac deaths [18, 37], with increased numbers typically observed in the morning between 06:00 a.m. and 12:00 p.m. [38].

Disruptions of the molecular clock have also been shown to contribute to cardiovascular disease. Knockout of the BMAL1 gene causes dilated cardiomyopathy in mouse models [39], and cardiac-specific deletion of BMAL1 initiates diastolic dysfunction, increases fibrotic responses, and impairs resolution of inflammation [40], thereby reducing survival from cardiomyopathy [41]. BMAL1 gene deletion in cardiomyocytes also impacts cardiac ion homeostasis that is important in the electrical activity of the heart by ablating circadian expression of Na+ and K+ channels, which could lead to sudden cardiac death [42, 43]. Together, these data indicate that circadian rhythms play an important role in structuring cardiovascular disease risk. The mechanism by which circadian rhythms generate time-of-day susceptibility to acute cardiovascular events remains to be revealed. However, due to circadian rhythms in the biomarkers used to assess cardiovascular disease (i.e., blood pressure, lipid panels, etc.), it is important to control for time-of-day when evaluating such clinical biomarkers. Additionally, since some exposures may vary in presence and intensity throughout the day-night cycle (e.g., air pollution), the interplay between cycles in exposure and circadian rhythms should also be considered.

### Breast Cancer

The disease for which circadian rhythms have been most intensely studied in environmental health is breast cancer. In 2006, using data from The Nurses’ Health Study II, which included over 100,000 female nurses from the U.S., it was discovered that women who reported 20+ years of night shift work had a relative risk of breast cancer of 1.79 compared to women who did not report shift work [44•]. It has been hypothesized that circadian disruption caused by shift work is responsible for elevated risk. There is now further evidence implicating circadian disruption in breast cancer risk; specifically, women in the Nurses’ Health Study II living in the most highly light polluted areas in the U.S. had a 14% higher risk of breast cancer compared to women living in the least light polluted areas [45]. A recent case-control study in Spain also found that both breast cancer and prostate cancer risk were associated with artificial light-at-night in the blue light spectrum, with blue being the most important part of the spectrum for circadian rhythms [46]. It is hypothesized that light pollution in cities contributes to light-at-night exposure on the individual level; therefore, light pollution can be used as a proxy for disruptive light-at-night exposure. The mechanisms by which light-at-night and circadian disruption may elevate cancer risk remain unknown; however, it may be related to the disruption of rhythms in the neuroendocrine and/or immune system that, when intact, may confer protection.

There are many limitations to current epidemiological studies of circadian rhythms and cancer. Light-at-night exposure and circadian disruption are difficult to measure, and there are potentially many confounding variables associated with shift work, light-at-night, and cancer. One recent study of over 100,000 women in the United Kingdom did not find consistent associations between breast cancer risk and self-reported night shift work in the past 10 years [47]. Taken together, the studies of cancer and circadian disruption highlight the need to develop protocols for evaluating circadian rhythm disruption as a risk modifier for diseases with environmental influence, such as breast cancer. These studies also highlight the need for including light pollution in the portfolio of environmental exposures that may impact health.

## Circadian Control of Clinical and Biological Biomarkers in Environmental Health

Environmental exposures have the ability to modify biological processes and mechanisms that contribute to downstream health and disease risks. At the same time, many of these biomarkers vary throughout the day-night cycle and have the potential to make individuals more or less vulnerable to environmental insults depending on the time of exposure. Here, we briefly discuss various processes known to have circadian rhythms and explore the mechanisms that link these processes to environmental exposures and health. We focus on five types of biological processes that are often used as markers of environmental exposures: oxidative stress, inflammation, endocrine disruption, DNA methylation, and histone modifications, all of which are important in epigenetic research and studies of exposure-related disease risk [48].

### Oxidative Stress

Various environmental pollutants are able to generate free radical reactions that result in oxidative damage. Environmental exposures, such as UV light or chemical pollutants, can lead to the production of reactive oxygen species (ROS), which can result in tissue injury, DNA damage, and cell death [49]. Protective enzymes and small-molecule antioxidants can counteract the detrimental effects of ROS that would otherwise leave cells in a prolonged state of oxidative stress [50].

Oxidative stress is often measured by assessing DNA damage, lipid peroxidation, and protein oxidation, all three of which have been shown to display circadian rhythms. It has been suggested that rhythms in oxidative stress biomarkers are due to rhythms in the response to oxidative stress, as opposed to rhythms in exposure, because these oscillations parallel the daily activity rhythms of protective enzymes and antioxidants [51]. Rodent models have been used in the study of oxidative stress rhythms. In rats, superoxide dismutases, which catalyze the dismutation of O_2_− into the less reactive species O_2_ and H_2_O_2_, have been shown to peak at night in the cerebral cortex [52]. Additionally, daily oscillations of glutathione (GSH), an antioxidant that removes hydroperoxides and neutralizes ROS, peaks in the middle of the light phase in rats and is inversely related to lipid peroxidation, suggesting that a decrease in GSH activity during the night could be partly responsible for the peak in lipid peroxidation at night [52]. Various other antioxidants and protective enzymes have also been shown to undergo daily redox cycles [53, 54].

In addition to the characterization of oxidative stress rhythms in humans and rodent models, studies in circadian clock mutants of Drosophila have demonstrated circadian control of antioxidant expression. In Drosophila, ROS levels, protein carbonylation, and mortality were significantly higher in wild-type flies exposed to H_2_O_2_-induced oxidative stress during the day versus the night, and this daily susceptibility rhythm was abolished in flies with a null mutation in the clock gene PER [55]. Additionally, mice deficient in the clock gene BMAL1 had an upregulation of ROS levels in several tissues. There is evidence that the circadian clock is important for ROS homeostasis and the aging process because the upregulation of ROS was associated with accelerated aging, and the administration of the antioxidant N-acetyl-L-cysteine ameliorated the premature aging [56, 57]. Taken together, studies of circadian rhythms in oxidative stress responses reveal that environmental health studies of oxidative stress should not only account for time-of-day when evaluating biological biomarkers, but could also evaluate circadian rhythms of participants in longer term studies of oxidative stress, DNA damage, and aging.

### Inflammation

Over the last decade, studies of circadian rhythms in the mammalian immune system have flourished. There are very well documented, self-sustained rhythms in the immune system driven by clock genes expressed within cells of the immune system. Because these rhythms are controlled by gene expression outside of the master clock in the SCN, the clock of the immune system is considered to be a peripheral clock. One of the earliest discoveries in this area was that leukocytes display circadian trafficking throughout the body. For instance, in mice—which are nocturnal and whose rhythms are expected to be phase shifted from diurnal mammals like humans—leukocytes migrate from the blood and enter into tissues at night. This trafficking has been shown to involve signals from the autonomic nervous system [58] and can directly impact the outcome of inflammatory diseases [59]. Particularly, circadian control of the immune system can dictate the timing of inflammatory disease onset [60, 61]. For example, rheumatoid arthritis patients exhibit increased joint inflammation, pain, and stiffness in the early morning hours, which results from nighttime synthesis and release of proinflammatory cytokines and chemokines, leading to increased cell migration to inflamed tissues during this time [62].

Circadian rhythms in the immune system and inflammatory response influence the intensity and onset of inflammation triggered in response to environmental stimuli. Exposure to environmental contaminants, such as allergens and air pollution, has the ability to cause inflammation; therefore, underlying rhythms in inflammation have the potential to modify the effect of inflammatory exposures. It is therefore necessary to understand the circadian regulation of inflammatory pathways in order to understand the pathophysiology of inflammatory diseases and appropriately time treatment.

### Endocrine Disruption

Endocrine-disrupting chemicals (EDCs), such as pesticides, flame retardants, plastics, and pharmaceuticals, are mostly man-made chemicals released into the environment that can interfere with the body’s hormonal signaling pathways and cause adverse health effects [63]. EDCs can alter the normal functioning of the endocrine system by mimicking natural hormones in the body, blocking the interaction of hormones with their receptors, or interfering with the way hormones or their receptors are made [64]. Certain classes of EDCs, including phthalates, dichlorodiphenyltrichloroethane (DDT), and bisphenol A (BPA), can affect reproductive health by mimicking or blocking the effects of male and female sex hormones [65]. For instance, interquartile range increases in the phthalate di-2-ethylhexyl phthalate (DEHP) detected in urine are associated with a 7.84% decrease in serum testosterone in young men and a 24.0–34.1% decrease in boys [66]. Due to the long recognized circadian rhythm in testosterone, models in such studies adjust for time-of-day when measuring the EDC effect on testosterone. In addition to testosterone, many hormones have been shown to have circadian rhythms in production.

In men, plasma testosterone peaks in the morning around 08:00 a.m. and decreases to its lowest concentrations in the evening [67, 68]. Total, free, and bioavailable testosterones are on average 30–35% higher at 08:00 a.m. compared to mid and late afternoon levels in young men [69]. The amplitude of the circadian rhythm declines with age, dropping to only a 10% difference between morning and late day in men 70 years of age [69]. The strong circadian rhythm in testosterone highlights the importance of accounting for rhythms when evaluating the effect of an environmental exposure, or for evaluating a biomarker in individuals of different ages. For example, early studies investigating the relationship between serum testosterone levels and age found inconsistent results [70–73], likely due to differences in blood sampling time; however, when controlling for sampling hour, an age-related decrease in testosterone levels was found in blood taken in the morning in young versus older men, but not in samples taken in the afternoon, which resulted from the loss of circadian rhythmicity in testosterone with aging [68]. With a 30–35% higher plasma testosterone level in the morning verses late day in young men, and a 7.84–34.1% decrease in urinary testosterone in boys and young men exposed to DEHP, it is clear that the natural daily variation in testosterone is comparable to the level of variation due to the environmental exposure, thus requiring the time-of-day adjustment in models.

The female reproductive hormones progesterone, estradiol, luteinizing hormone (LH), and follicular stimulating hormone (FSH) also have circadian rhythms and, importantly, vary across the menstrual cycle [74]. For instance, during the follicular phase of the menstrual cycle, estradiol exhibits a peak around bedtime, progesterone displays an early morning peak after waking, and FSH levels wane at night and climb during the day, reaching a peak before bedtime [74]. The circadian rhythms of female reproductive hormones are therefore aligned to have a particular phase position such that they are not all peaking at the same time-of-day. Nocturnal FSH levels have been shown to decline by 17–18% compared to daytime values [75], and FSH, progesterone, and LH all have similar circadian amplitude [74]. Like testosterone, the circadian variation in female reproductive hormones is comparable to the level of variation observed in response to environmental exposures. For example, a 10% decrease in mean FSH has been reported for cadmium exposures at the highest compared to lowest exposed tertile [76]. Given the variability observed in hormone levels throughout the day, not only do the effects of EDCs on hormones need to account for time-of-day, but the effect of EDCs on the rhythms themselves could be studied. For instance, could EDCs alter the amplitude or phase of hormone rhythms? Or do EDCs merely adjust the mean?

### DNA Methylation

The study of DNA methylation is currently a major area of Environmental Health Sciences. Environmental pollutants have been linked to disease phenotypes through epigenetic modifications such as DNA methylation, histone modifications, and microRNAs [77]. DNA methylation occurs post-DNA replication and involves the addition of a methyl group at the fifth carbon position of cytosine residues, which leads to changes in gene expression without changing the underlying DNA sequence. Aberrant DNA methylation has been observed in various cancer types [78], autoimmune diseases [79], metabolic disorders [80], and aging [81]. Methylation analysis therefore has the potential to aid in the diagnosis and treatment of various diseases [82].

In contrast to the prevailing view that DNA methylation is a relatively stable epigenetic marker, recent evidence suggests that DNA methylation can fluctuate daily and seasonally [83•, 84]. Levels of DNA methylation in a number of gene promoter regions have been shown to display circadian changes [85, 86]. In mice, expression of enzymes involved in DNA methylation and demethylation in the SCN can be induced by light in a circadian phase-dependent manner, which could be a way that the SCN drives circadian clock plasticity [83•]. Daily cycles in DNA methylation have also been demonstrated in the blood of healthy individuals, with increased levels of DNA methylation occurring at night [87]. Work in human cells lines has revealed that DNA methylation sites can fluctuate within a single cell cycle [85, 86, 88], thereby demonstrating the dynamic nature of DNA methylation. Although one may assume daily cycles in DNA methylation could be driven by daily cycles in cell proliferation and associated DNA replication, slow proliferating cells of the liver, lung, and brain in mice also exhibit daily cycles in methylation, particularly at CpG sites [89–91]. These circadian oscillations of cytosine modifications are also prevalent in human neutrophils and are associated with complex diseases including leukemia, schizophrenia, and diabetes [92].

### Histone Post-translational Modifications

Histones are nuclear globular proteins that undergo posttranslational modifications such as acetylation, methylation, phosphorylation, SUMOylation, ribosylation, and ubiquitination [93]. Histones alter chromatin structure and hence modulate gene expression and genome stability [94, 95]. Environmental pollutants, such as pesticides and metals, can affect histone modifications and/or alter the function of enzymes involved in histone modifications [96, 97]. For example, arsenic exposure can inhibit histone deacetylases and induce chromatin opening by histone hyperacetylation [98]. Aberrant patterns of histone posttranslational modifications in the genome have been associated with various human diseases, including autoimmune diseases [99], neurodegenerative diseases [100], and cancer [101], and thus represent an important epigenetic alteration in environmental diseases.

A direct link between the circadian clock and epigenetics via histone modifications can be found in the CLOCK gene, which has been shown to possess intrinsic histone acetyltransferase activity that is necessary for circadian gene expression [102]. CLOCK also acetylates non-histone substrates, such as BMAL1, in a circadian manner [103], suggesting that other targets in the body may also be affected. Indeed, CLOCK can also acetylate the glucocorticoid receptor, thereby attenuating its DNA binding and regulating its function [104, 105]. The activation of several clock-controlled genes, including PER1, PER2, and Dbp, has also been associated with rhythmic changes in histone acetylation at their promoters [106–108]. These studies suggest that one way in which the circadian clock exerts transcriptional control is through mechanisms involving histone modification and chromatin remodeling. Daily alterations in these factors have been linked to circadian changes in processes such as metabolism and cellular proliferation [109, 110]. The study of circadian histone modification has largely focused on the expression of clock genes; importantly, however, mouse models have also shown that 43% of all protein coding genes have circadian expression somewhere in the body (e.g., liver, kidney, lung, brown fat, etc.) [111•]; therefore, circadian histone modification could be impacting the expression of genes in addition to the core clock genes.

## Conclusion

In conclusion, circadian rhythms are a main structuring feature of the human body and exist at multiple levels of organization from molecular to physiological processes. Circadian rhythms impact molecular processes within cells, such as responses to oxidative stress, DNA replication, DNA methylation, histone modification, and gene expression. As for cellular and physiological processes, circadian rhythms drive cell trafficking in the immune system and the production of neuroendocrine and reproductive hormones. We suggest that all studies should aim to control for circadian rhythms when selecting biomarkers of exposure or health. Biological processes and biomarkers vary in the degree to which they display rhythmicity, and those that are rhythmic vary in their amplitude of circadian variation. When measuring the effect of exposures on biological processes, it is important to know the magnitude of circadian variation relative to the amount of variation due to environmental exposures. Lack of controls for circadian rhythms could mask important variation due to exposures.

When considering exposures such as air pollution, endocrine-disrupting chemicals, etc., it is important to recognize that the time-of-day of exposure could impact health risk. For example, exposure to artificial light at night can impact biological processes and have pathological consequences, whereas artificial light exposure during the day would not have the same effect [12•]. Given that the circadian clock is entrained by light, studies should acknowledge that the rhythmicity of certain biomarkers may be affected when sampled under varying lighting conditions, such as in the winter (short days) vs. summer (long days) or for people who spend most of their time indoors (under artificial light) vs. outdoors (under natural light). Ideally, but often unfeasible, study designs should deploy circadian and time-dependent sampling, with multiple samples being collected at regular intervals across several days. Such longitudinal sampling allows researchers to better assess within-individual changes over time and can indicate whether we are missing important variation in physiological and behavioral responses. For instance, this can be accomplished through the collection of at least three sampling timepoints within a 24-h period: shortly after waking, midday, and immediately before bed. If this is not feasible and a study is limited to the collection of a single sample, researchers should control for the time in which the sample is collected or include that information in the subsequent statistical analyses.

In this review, we have highlighted the importance of circadian rhythms in environmentally influenced diseases such as asthma, breast cancer, and heart disease. We believe that the intersection of circadian biology and environmental health offers a novel angle for exploring the mechanism of disease pathology and susceptibility. In particular, one interesting avenue for future research is determining how circadian rhythms may influence individual susceptibility to environmental exposures throughout the day. For instance, would the same dose of PM_2.5_ air pollution have the same effect on the body in the morning as the evening? If there is circadian variation in susceptibility, should this be taken into account in air pollution policy? Studies could also evaluate how light-at-night, which causes circadian rhythm disruption, interacts with other environmental exposures such as noise and air pollution, potentially magnifying health effects. Lastly, the majority of best-selling drugs and essential medicines target the product of circadian genes [111•]. This alone demonstrates the importance of acknowledging rhythms in the overall science of health, as we seek to improve treatment and prevention.
